# Prognostic Value of Serum MicroRNA-423-5p Levels for Cardiovascular Events in Patients with Heart Failure

**DOI:** 10.3390/biomedicines14061319

**Published:** 2026-06-10

**Authors:** Tuong Trong Le Huynh, Son Kim Tran

**Affiliations:** 1 Department of Internal Medicine, Faculty of Medicine, Can Tho University of Medicine and Pharmacy, 179 Nguyen Van Cu Street, Tan An Ward, Can Tho City 900000, Vietnam; huynhletrongtuong@gmail.com; 2 Department of Cardiology, Cardiovascular Center, Can Tho Central General Hospital, 315 Nguyen Van Linh Street, Tan An Ward, Ninh Kieu District, Can Tho City 900000, Vietnam

**Keywords:** heart failure, prognosis, cardiovascular events, microRNA-423-5p level

## Abstract

**Background**: Patients with heart failure remain at a relatively high risk of cardiovascular events after hospital discharge, adversely affecting survival and quality of life. Recent evidence suggests that serum microRNA-423-5p may serve as a prognostic biomarker; however, data in Vietnamese populations are limited. **Objectives**: To evaluate the prognostic value of serum microRNA-423-5p for major adverse cardiovascular events (MACE) in patients with heart failure. **Materials and Methods**: A cross-sectional study with a 6-month follow-up was conducted in 250 heart failure patients at Can Tho Central General Hospital from January 2024 to November 2025. Serum microRNA-423-5p levels were measured, and patients were followed for MACE, including rehospitalization for heart failure, non-fatal myocardial infarction, cardiovascular death, and all-cause mortality. **Results**: Males accounted for 56.0%. Patients with LVEF < 30% had higher NT-proBNP levels and smoking prevalence compared to those with LVEF ≥ 30% (*p* < 0.05). At 6 months, 29.6% experienced MACE, most commonly heart failure rehospitalization (23.2%), followed by all-cause mortality (7.6%), cardiovascular death (6.0%), and myocardial infarction (1.6%). Median microRNA-423-5p levels were higher in patients with MACE [4.23 (IQR: 1.82–17.63) vs. 2.50 (IQR: 0.96–5.46), (2^−ΔCt^); *p* < 0.05]. A cutoff value of 10.77 (2^−ΔCt^) demonstrated moderate prognostic performance (AUC = 0.652; 95% CI: 0.574–0.729; *p* < 0.05). Multivariable analysis identified LVEF < 30% (OR = 1.94) and microRNA-423-5p (OR = 1.14) as independent predictors of MACE. **Conclusions**: Serum microRNA-423-5p is a useful biomarker for predicting 6-month cardiovascular events in patients with heart failure.

## 1. Introduction

Heart failure represents the final common pathway of various cardiovascular diseases and has become increasingly prevalent due to population aging and advances in cardiovascular therapies, including valve surgery, coronary interventions, ventricular assist devices, and guideline-directed medical therapies such as SGLT2 inhibitors. However, mortality rates remain high [1,2]. Globally, approximately 1–2% of the adult population is affected by heart failure. In low- and middle-income countries, heart failure has been identified as the fastest-growing cardiovascular condition [3]. Patients with heart failure remain at substantial risk of developing cardiovascular events after hospital discharge, which significantly reduce both quality of life and life expectancy. Therefore, identifying predictors of these adverse outcomes is of critical importance.

In addition to traditional biomarkers such as NT-proBNP and LVEF, recent studies have suggested that serum microRNA-423-5p may have prognostic value for cardiovascular events in heart failure patients [4]. MicroRNA-423-5p is a mature miRNA derived from the *MIR423* gene and is expressed in cardiac tissue, where it is associated with myocardial function. Goldraich et al. reported that circulating microRNA-423-5p originates directly from the myocardium in patients with heart failure [5]. This miRNA is involved in signaling pathways and gene expression related to myocardial injury. Luo et al. demonstrated that microRNA-423-5p directly targets O-GlcNAc transferase (OGT), a key enzyme in protein O-GlcNAcylation. Upregulation of microRNA-423-5p may influence OGT activity and activate apoptosis pathways in cardiomyocytes, thereby contributing to myocardial damage [6]. Emerging evidence has also explored its prognostic role; for instance, Schneider et al. reported that during follow-up, 79% of patients required rehospitalization at least once and 44% died, with dynamic changes in plasma microRNA-423-5p associated with clinical improvement and prognosis [7]. Similarly, a study in China found that microRNA-423-5p was positively correlated with NT-proBNP (r = 0.609, *p* < 0.05) and negatively correlated with LVEF (r = −0.406, *p* < 0.05). Furthermore, its levels were shown to change in response to dapagliflozin therapy, suggesting a potential role in prognostic assessment of heart failure [8]. However, data regarding the prognostic value of microRNA-423-5p in patients with heart failure in Southeast Asian countries, particularly in the Vietnamese population, remain limited. Furthermore, the role of this biomarker in short-term risk stratification among hospitalized patients with heart failure in routine clinical practice has not been fully clarified. Therefore, the present study was conducted to evaluate the prognostic value of serum microRNA-423-5p levels for predicting 6-month cardiovascular events in a Vietnamese population of patients with heart failure.

## 2. Materials and Methods

### 2.1. Study Design and Population

A prospective observational cohort study with a 6-month follow-up was conducted in hospitalized patients with heart failure with reduced ejection fraction (HFrEF) at Can Tho Central General Hospital from January 2024 to November 2025. The diagnosis of heart failure was based on the European Society of Cardiology criteria, requiring the simultaneous presence of: (1) symptoms of heart failure; (2) reduced left ventricular ejection fraction (LVEF) on echocardiography; and (3) elevated NT-proBNP levels. Exclusion criteria included: (1) patients with malignancy; (2) severe renal or hepatic failure; (3) patients with psychiatric disorders or dementia; and (4) patients lost to follow-up or those who continued follow-up at other healthcare facilities. Patients with incomplete clinical or laboratory data were also excluded from the final analysis. Only patients with complete clinical, laboratory, and follow-up data were included in the final statistical analysis. At the end of the follow-up period, a total of 250 eligible patients were enrolled and included in the final analysis [9].

### 2.2. Data Collection

Patients’ anthropometric characteristics were recorded, and body mass index (BMI) was classified according to Asian-specific criteria [10]. Clinical examinations were performed by specialist physicians to document heart failure symptoms and comorbidities, including hypertension, diabetes mellitus, and dyslipidemia. A family history of premature cardiovascular disease was defined as the presence of cardiovascular events (myocardial infarction or sudden cardiac death) in first-degree relatives (parents, siblings, or offspring) before the age of 55 years in men or 65 years in women. Eleven serum microRNA-423-5p levels were measured at the time of hospital admission. Peripheral venous blood (4 mL) was collected from each participant, temporarily stored at 4 °C, and promptly transported to the Molecular Biology Center for processing. Blood samples were centrifuged at 5000 rpm for 15 min at room temperature to obtain serum. Following extraction, microRNA-423-5p levels were quantified using quantitative polymerase chain reaction (qPCR) with the miRCURY LNA PCR Assay (Qiagen, Hilden, Germany). All qPCR procedures were performed in accordance with the manufacturer’s standard protocol. The assay is based on the use of specific primers for microRNA-423-5p, optimized with Locked Nucleic Acid (LNA) technology.

Serum microRNA-423-5p levels were measured at the time of hospital admission. Peripheral venous blood (4 mL) was collected from each participant, temporarily stored at 4 °C, and promptly transported to the Molecular Biology Center for processing. Blood samples were centrifuged at 5000 rpm for 15 min at room temperature to obtain serum. Serum samples were subsequently stored under appropriate laboratory conditions until RNA extraction.

Following extraction, microRNA-423-5p levels were quantified using quantitative polymerase chain reaction (qPCR) with the miRCURY LNA miRNA PCR Assay (Qiagen, Hilden, Germany). All qPCR procedures were performed according to the manufacturer’s standard protocol. The assay utilizes Locked Nucleic Acid (LNA)-enhanced primers specific for microRNA-423-5p. Relative expression levels were calculated using the 2^−ΔCt^ method. Samples with inadequate quality for analysis were excluded according to standard laboratory procedures.

### 2.3. Follow-Up Outcomes

All patients received treatment according to European Society of Cardiology (ESC) guidelines, including guideline-directed medical therapy with the four foundational drug classes: SGLT2 inhibitors, beta-blockers, renin–angiotensin system inhibitors (ACE inhibitors/ARBs/ARNI), and mineralocorticoid receptor antagonists. After discharge, patients were followed for cardiovascular events during hospitalization and at 6 months post-discharge. Major adverse cardiovascular events (MACE) were defined as a composite of rehospitalization for heart failure, non-fatal myocardial infarction, cardiovascular death, and all-cause mortality. Follow-up was conducted through direct clinical visits at monthly outpatient appointments, review of discharge summaries and prescription records, and telephone interviews with patients or their relatives. All reported events were further verified using electronic medical records. Collected data included the type of event and time to event occurrence. At the end of the follow-up period, all data were compiled and subjected to statistical analysis.

### 2.4. Statistical Analysis

Data were analyzed using SPSS software version 20.0 (IBM Corp., Armonk, NY, USA). Categorical variables were presented as frequencies (percentages), while continuous variables were expressed as mean ± standard deviation (SD) for normally distributed data and median (interquartile range, IQR) for non-normally distributed data. Normality was assessed using the Kolmogorov–Smirnov test and was considered satisfied when the significance level (Sig.) was greater than 0.05. Differences between categorical variables were evaluated using the Chi-square test or Fisher’s exact test, as appropriate. Comparisons of medians between two or more groups were performed using the Kruskal–Wallis test. Receiver operating characteristic (ROC) curve analysis was applied to determine the optimal cutoff value of serum microRNA-423-5p for predicting outcomes.

### 2.5. Ethical Approval

All participants were informed about the study objectives and methods and were free to withdraw at any stage of the study. All collected information was used solely for research purposes and was not disclosed without the participants’ consent. The study protocol was approved by the Institutional Review Board of Can Tho University of Medicine and Pharmacy and was conducted in accordance with the principles of the Declaration of Helsinki (Approval No.: 23.016.NCS/PCT-HĐĐĐ).

## 3. Results

Among 250 patients with heart failure (Table 1), males accounted for 56.0% of the study population. Patients with LVEF < 30% had a higher median NT-proBNP level compared to those with LVEF ≥ 30% (7678.80 vs. 3592.00 pg/mL). Similarly, the prevalence of smoking was higher in the LVEF < 30% group (64.2% vs. 42.6%) (*p* < 0.05).

After 6 months of follow-up (Table 2), 29.6% of patients experienced major adverse cardiovascular events (MACE). The most common event was rehospitalization for heart failure (23.2%), followed by all-cause mortality (7.6%), cardiovascular mortality (6.0%), and acute myocardial infarction (1.6%).

The study found that patients who experienced MACE after 6 months of follow-up had a higher median microRNA-423-5p level compared to those without MACE [4.23 (IQR: 1.82–17.63) vs. 2.50 (IQR: 0.96–5.46), *p* < 0.05] (Table 3).

**Figure 1:**
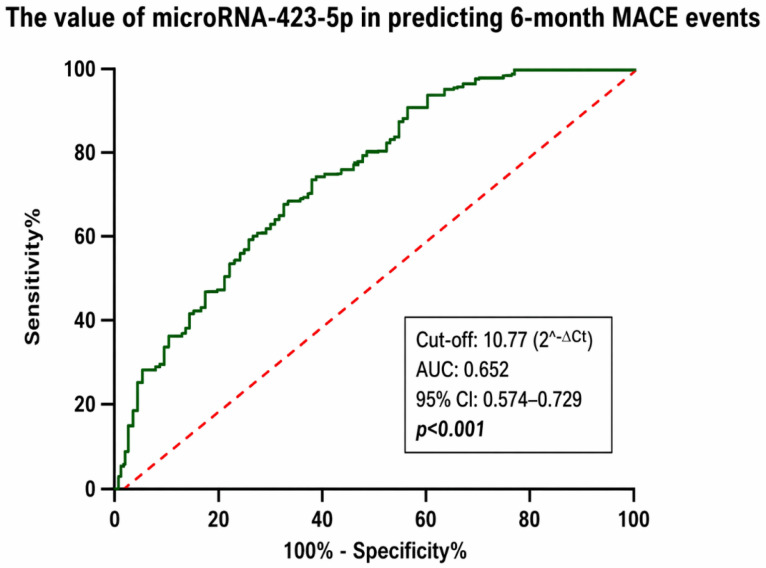
Prognostic value of microRNA-423-5p for predicting MACE at 6 months. Receiver operating characteristic (ROC) curve analysis of serum microRNA-423-5p levels for predicting major adverse cardiovascular events (MACE) at 6 months in patients with heart failure. The area under the curve (AUC) was 0.652 (95% CI: 0.574–0.729; *p* < 0.001).

MicroRNA-423-5p at a cutoff value of 10.77 (2^−ΔCt^) demonstrated moderate prognostic value for predicting MACE at 6 months, with an AUC of 0.652 (95% CI: 0.574–0.729; *p* < 0.05) (Figure 1).

**Figure 2:**
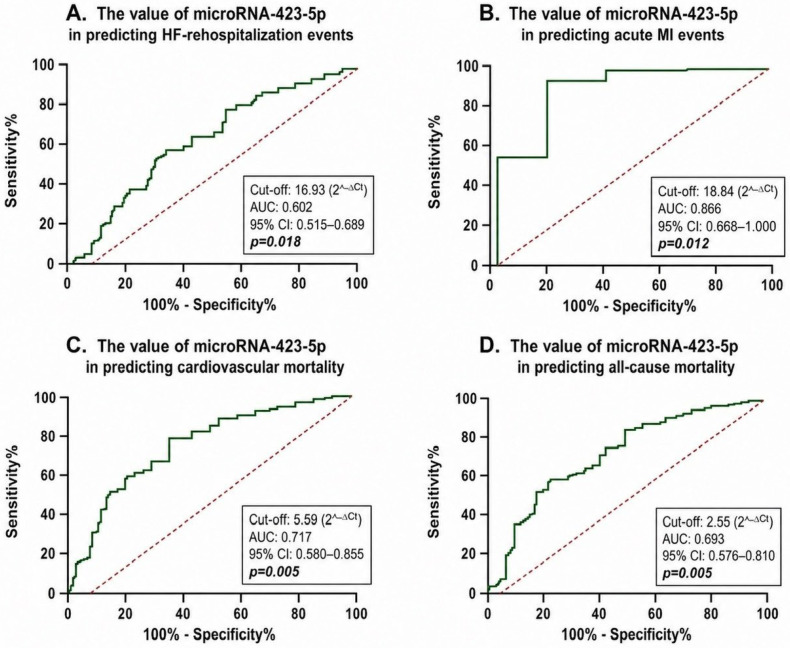
Prognostic value of microRNA-423-5p for predicting individual component events at 6 months. Receiver operating characteristic (ROC) curve analyses of serum microRNA-423-5p levels for predicting individual cardiovascular outcomes at 6 months in patients with heart failure. (**A**) Rehospitalization for heart failure. (**B**) Acute myocardial infarction. (**C**) Cardiovascular mortality. (**D**) All-cause mortality.

When analyzing individual component events, microRNA-423-5p also demonstrated moderate prognostic value for predicting rehospitalization for heart failure (AUC = 0.602), myocardial infarction (AUC = 0.866), cardiovascular death (AUC = 0.717), and all-cause mortality (AUC = 0.693) (Figure 2).

In univariable analysis, coronary artery disease (OR = 3.78), family history of cardiovascular disease (OR = 2.60), LVEF < 30.0% (OR = 1.82), and microRNA-423-5p (OR = 1.13) were associated with MACE. However, in multivariable analysis, only two factors—LVEF < 30.0% (OR = 1.94) and microRNA-423-5p (OR = 1.14)—remained independently associated with the prediction of MACE at 6 months (*p* < 0.05) (Table 4).

## 4. Discussion

Despite significant advances in treatment, patients with heart failure remain at a high risk of cardiovascular events. The rehospitalization rate within one year ranges from 10% to 30%, with more than 90% of admissions attributable to symptoms and signs of congestion [11]. In addition, over one-quarter of patients (24%) are rehospitalized within 30 days, and the rehospitalization rate within the first 3 months may reach up to 30% [12]. Therefore, predicting cardiovascular events in this population is of critical importance. According to Andrew H. Coles et al., an analysis of data from 11 medical centers in central Massachusetts showed that 35% (n = 1414) of patients had reduced ejection fraction, 13% (n = 521) had mildly reduced ejection fraction (41–49%), and 52% (n = 2090) had preserved ejection fraction (≥50%). At one year after discharge, mortality rates were 34%, 30%, and 29%, respectively (*p* = 0.03) [13]. Similarly, all-cause mortality was reported to be relatively high (29%) in the study by Alain Rudiger. Overall, 28% of patients had newly diagnosed heart failure, while 72% experienced acute decompensation, with coronary artery disease being the most common etiology. Cardiogenic shock occurred in 4% and pulmonary edema in 13% of patients. The 12-month all-cause mortality rate (29%) increased in the presence of shock, left ventricular dysfunction, renal failure, coronary artery disease, and advanced age [14]. In another study, Carlo Lombardi et al. evaluated all-cause and cardiovascular mortality as primary endpoints. Among 728 patients, 256 deaths were recorded, including 142 due to cardiovascular causes. The 1-year mortality rate was 20%, with the highest risk observed during hospitalization (approximately 8% at 30 days). The risk of adverse events was higher during hospitalization, particularly for cardiovascular mortality [15]. Sun et al. conducted a study in China involving 468 patients with chronic heart failure followed for 6 months after discharge. The primary endpoint (MACE), including cardiovascular death, rehospitalization for heart failure, non-fatal myocardial infarction, and non-fatal stroke, occurred in 33.3% of patients [16]. A clinical study in India reported a 90-day mortality rate of 14.2% (14.9% in females and 13.9% in males) and a rehospitalization rate of 8.4% [17]. These findings are consistent with our results, which showed that after 6 months of follow-up, 29.6% of patients experienced MACE. The most common event was rehospitalization for heart failure (23.2%), followed by all-cause mortality (7.6%), cardiovascular mortality (6.0%), and acute myocardial infarction (1.6%).

Our study demonstrated that serum microRNA-423-5p, at a cut-off value of 10.77 (2^−ΔCt^), showed good prognostic performance for major adverse cardiovascular events (MACE). Notably, changes in microRNA-423-5p levels were identified as an independent predictor of MACE, alongside left ventricular ejection fraction (LVEF), suggesting its incremental value in risk stratification. These findings are consistent with previous evidence indicating that heart failure is a complex syndrome influenced not only by hemodynamic impairment but also by underlying metabolic disturbances. Son et al. (2022) reported that even in non-diabetic and non-obese patients with heart failure, prediabetes and insulin resistance were highly prevalent and associated with elevated NT-proBNP levels, reduced LVEF, and more severe disease [18]. This suggests that emerging biomarkers such as microRNA-423-5p may reflect both myocardial injury and underlying metabolic dysregulation contributing to adverse outcomes. Similarly, a meta-analysis by Reza Parvan et al., including 22 studies with 5736 patients, identified several microRNAs as predictors of cardiovascular outcomes. Among these, microRNA-423-5p, together with microRNA-122-5p, was significantly associated with cardiovascular mortality in patients with heart failure with reduced ejection fraction (HFrEF), with a hazard ratio (HR) of 3.61 (95% CI: 2.67–4.87) [19]. In addition, a systematic review and meta-analysis conducted by Jie Yang et al., covering studies published between 2010 and 2018, demonstrated that microRNA-423-5p, along with microRNA-30 and microRNA-18, was significantly associated with overall survival in heart failure patients. Notably, lower circulating levels of microRNA-423-5p were associated with worse prognosis and higher mortality risk [20]. Taken together, these findings further support the role of microRNA-423-5p as a promising novel biomarker for predicting cardiovascular outcomes in patients with heart failure.

Furthermore, recent evidence suggests that the relationship between LVEF and prognosis in patients with heart failure may not be entirely linear. Although severely reduced LVEF remains strongly associated with adverse cardiovascular outcomes, several contemporary studies have demonstrated that supranormal LVEF values may also be associated with an increased risk of mortality. These findings challenge the traditional concept that higher LVEF is uniformly protective and further highlight the marked heterogeneity of heart failure phenotypes [21,22]. In this context, emerging biomarkers such as microRNA-423-5p may provide additional prognostic information beyond conventional structural and functional parameters, thereby contributing to improved individualized risk stratification in patients with heart failure.

Although several previous studies have reported associations between circulating microRNA-423-5p and outcomes in patients with heart failure, data from Southeast Asian populations, particularly the Vietnamese population, remain limited. The present study therefore provides additional region-specific evidence regarding the prognostic role of microRNA-423-5p in hospitalized Vietnamese patients with heart failure. In addition, our findings reflect real-world clinical practice by evaluating short-term cardiovascular outcomes during the 6-month post-discharge period. Beyond demonstrating an association between microRNA-423-5p and MACE, the present study also evaluated its prognostic performance through ROC curve and multivariable regression analyses, in which microRNA-423-5p remained independently associated with adverse cardiovascular outcomes after adjustment for relevant clinical factors. These findings further support the potential utility of microRNA-423-5p as a complementary biomarker for risk stratification in patients with heart failure.

This study has several strengths. First, it is among the few studies conducted in a Vietnamese population evaluating the prognostic value of microRNA-423-5p in patients with heart failure, thereby providing region-specific clinical evidence. Second, the prospective observational design with clearly defined outcomes enhances the reliability of event ascertainment. Third, the use of standardized laboratory techniques (qPCR) and guideline-directed management according to ESC recommendations improves the consistency and clinical relevance of the findings. In addition, multiple methods were employed to verify outcomes, including direct clinical visits, medical record review, and telephone follow-up, thereby minimizing information bias. However, several limitations should be acknowledged. This was a single-center study with a relatively modest sample size, which may limit the generalizability of the findings. The follow-up duration of 6 months, although clinically meaningful, may not fully capture long-term outcomes. Serum microRNA-423-5p levels were measured only at baseline, and dynamic changes over time were not assessed. Furthermore, despite multivariable adjustment, residual confounding from unmeasured variables cannot be excluded. Although microRNA-423-5p remained independently associated with MACE in multivariable analysis, the present study was not specifically designed to evaluate its incremental prognostic value beyond established biomarkers such as NT-proBNP and LVEF. Future large-scale multicenter studies incorporating integrated predictive models are warranted to further clarify the additional prognostic value of microRNA-423-5p in clinical practice.

## 5. Conclusions

Patients with heart failure have a relatively high incidence of cardiovascular events at 6 months after hospital discharge, affecting nearly 30.0% of cases. Patients who experienced events had higher median levels of microRNA-423-5p compared to those without events. A cutoff value of 10.77 (2^−ΔCt^) demonstrated good prognostic value. Notably, LVEF < 30.0% and microRNA-423-5p were identified as independent predictors of cardiovascular events at 6 months.

## Figures and Tables

**Table 1:** Baseline characteristics of patients with heart failure included in the study.

Characteristics		Overall (n = 250)	LVEF Groups		*p*
			LVEF < 30% (n = 81)	LVEF ≥ 30% (n = 169)	
Male		140 (56.0)	57 (70.4)	83 (49.1)	0.002
Age (years)		68.15 ± 14.24	65.38 ± 15.51	69.48 ± 13.43	0.033
BMI (kg/m^2^)		21.86 ± 2.23	21.88 ± 2.22	21.85 ± 2.23	0.916
NYHA	Class II	26 (10.4)	7 (8.6)	19 (11.3)	0.758
	Class III	208 (83.2)	68 (84.0)	140 (82.8)	
	Class IV	16 (6.4)	6 (7.4)	10 (5.9)	
NT-proBNP (pg/mL)		4556.20 (1987.00–11,589.62)	7678.80 (2640.00–12,942.50)	3592.00 (1327.80–11,289.20)	0.001
MicroRNA-423-5p (2^−ΔCt^)		2.66 (1.11–6.96)	2.54 (1.08–5.60)	3.41 (1.12–7.32)	0.470
Smoking		124 (49.6)	52 (64.2)	72 (42.6)	0.001
Hypertension		224 (89.6)	71 (87.7)	153 (90.5)	0.485
Diabetes mellitus		49 (19.6)	16 (19.8)	33 (19.5)	0.966
Dyslipidemia		228 (91.2)	74 (91.4)	154 (91.1)	0.951
Coronary artery disease		200 (80.0)	70 (86.4)	130 (76.9)	0.079
Family history of CVD		165 (66.0)	57 (70.4)	108 (63.9)	0.313

**Table 2:** Incidence of cardiovascular events after 6 months of follow-up.

Events	Overall (n = 250)	LVEF Groups		*p*
		LVEF < 30% (n = 81)	LVEF ≥ 30% (n = 169)	
MACE	74 (29.6)	31 (38.3)	43 (25.4)	0.038
Rehospitalization for heart failure	58 (23.2)	22 (27.2)	36 (21.3)	0.304
Acute myocardial infarction	4 (1.6)	3 (3.7)	1 (0.6)	0.101
Cardiovascular mortality	15 (6.0)	4 (4.9)	11 (6.5)	0.780
All-cause mortality	19 (7.6)	8 (9.9)	11 (6.5)	0.347

**Table 3:** Comparison of microRNA-423-5p levels according to cardiovascular events after 6 months of follow-up.

Events		MicroRNA-423-5p (2^−ΔCt^)	*p*
MACE	Yes	4.23 (1.82–17.63)	<0.001
	No	2.50 (0.96–5.46)	
Rehospitalization for heart failure	Yes	3.77 (1.53–18.36)	0.018
	No	2.56 (1.01–6.17)	
Acute myocardial infarction	Yes	39.18 (6.99–169.53)	0.012
	No	2.64 (1.11–6.83)	
Cardiovascular mortality	Yes	7.39 (2.57–16.43)	0.005
	No	2.60 (1.06–6.17)	
All-cause mortality	Yes	5.68 (2.57–14.38)	0.005
	No	2.57 (1.05–6.17)	

**Table 4:** Logistic regression analysis of factors associated with MACE at 6 months of follow-up.

Variables		MACE at 6 Months		OR (95% CI) a	OR (95% CI) b
		Yes (n, %)	No (n, %)		
Smoking	Yes	38 (51.4)	86 (48.9)	1.11 (0.64–1.90)	-
	No	36 (48.6)	90 (51.1)		
Hypertension	Yes	70 (94.6)	154 (87.5)	2.50 (0.83–7.53)	-
	No	4 (5.4)	22 (12.5)		
Diabetes mellitus	Yes	13 (17.6)	36 (20.5)	0.83 (0.41–1.67)	-
	No	61 (82.4)	140 (79.5)		
Dyslipidemia	Yes	71 (95.9)	157 (89.2)	2.86 (0.82–9.99)	-
	No	3 (4.1)	19 (10.8)		
Coronary artery disease	Yes	68 (91.9)	132 (75.0)	3.78 (1.53–9.31) *	2.36 (0.72–7.72)
	No	6 (8.1)	44 (25.0)		
Family history of CVD	Yes	59 (79.7)	106 (60.2)	2.60 (1.37–4.94) *	1.72 (0.72–4.12)
	No	15 (20.3)	70 (39.8)		
LVEF < 30.0%	Yes	31 (41.9)	50 (28.4)	1.82 (1.03–3.20) *	1.94 (1.03–3.66) *
	No	43 (58.1)	126 (71.6)		
Age (years)		70.23 ± 13.32	67.28 ± 14.55	1.01 (0.99–1.04)	-
BMI (kg/m^2^)		21.73 ± 2.61	21.91 ± 2.05	0.96 (0.85–1.09)	-
MicroRNA-423-5p (2^−ΔCt^)		4.23 (1.82–17.63)	2.50 (0.96–5.46)	1.13 (1.07–1.19) *	1.14 (1.08–1.21) *
NT-proBNP (pg/mL)		5646.95 (2053.50–11,707.67)	3601.90 (1825.25–11,564.75)	1.00 (0.99–1.01)	-

a: Univariable analysis; b: Multivariable analysis; * *p* < 0.05.

## Data Availability

The datasets used and/or analyzed during the current study are available from the corresponding author upon reasonable request.

## Abbreviations

The following abbreviations are used in this manuscript:

BMI	Body mass index
CAD	Coronary artery disease
HF	Heart failure
LNA	Locked nucleic acid
LVEF	Left ventricular ejection fraction
MACE	Major adverse cardiovascular events
miRNA	MicroRNA
OGT	O-GlcNAc transferase
qPCR	Quantitative polymerase chain reaction
